# The Effect of Personality Traits on the Quality of Life of Health Professionals in COVID-19 Reference Hospital

**DOI:** 10.7759/cureus.17897

**Published:** 2021-09-12

**Authors:** Dimitra Lekka, Konstantina Orlandou, Aikaterini Roubi, Dimitra Darahani, Sofia Mpoulougari, Frosyna Anagnosti, Spyros Baras, Athanasios Tsaraklis, Anastasios Stalikas

**Affiliations:** 1 Department of Psychiatry, Sotiria Hospital, Athens, GRC; 2 Department of Psychology, Panteion University, Athens, GRC; 3 Department of Psychiatry, Sotiria Thoracic Diseases Hospital of Athens, Athens, GRC; 4 Department of Cardiology, Sotiria Thoracic Diseases Hospital of Athens, Athens, GRC; 5 Department of Administration, Sotiria Thoracic Diseases Hospital of Athens, Athens, GRC; 6 Emergency Department, Sotiria Thoracic Diseases Hospital of Athens, Athens, GRC; 7 Department of Dermatology, Sotiria Hospital, Athens, GRC

**Keywords:** quality of life, personality traits, hospital staff, covid-19 reference hospital, greece

## Abstract

Introduction and objectives

Coronavirus disease 2019 (COVID-19) has affected the quality of life of both general population and the healthcare workers and has increased the psychopathology levels. The objective of this research was to study the personality traits and the quality of life of healthcare professionals during the COVID-19 pandemic, in order to organize and apply interventions for the well-being of the staff.

Materials and methods

The study sample consisted of 400 healthcare workers, in Thoracic Diseases General Hospital "Sotiria". Participants were asked to provide sociodemographic information and to complete: (1) the WHOQOL-BREF, (2) the NEO-FFI. The questionnaire was administered in person to the hospital staff. The data were collected between May and July 2021, in Athens, Greece.

Results

Younger healthcare professionals had a better quality of life and with regard to gender differences, males reported higher scores of physical and psychological health compared to females. Also regarding personality traits, neuroticism and extroversion have a statistically significant effect on the quality of life. In contrast, education level, work area and specialty did not appear to affect the quality of life of hospital staff.

Conclusions

From our research findings, it appears that quality of life has been affected by the COVID-19 pandemic. Interventions are needed to apply mindfulness, increase well-being and form groups with the hospital staff in order to share their experiences.

## Introduction

In the end of December 2019, a virus named COVID-19 by WHO, was reported in Wuhan, China for the first time and spread rapidly around the world [1,2]. The declaration of the pandemic of COVID-19 produced big changes in healthcare professionals, because the main characteristic of the situation was the uncertainty. Scientific evidence has shown the impact of the pandemic and the confinement measures to psychopathology of healthcare workers, but we know little about the quality of hospital professionals' life [3-5].

Moderate and high levels of depression, post-traumatic stress and anxiety were observed, during the current COVID-19 crisis, in the frontline healthcare workers, who were particularly susceptible to developing psychiatric disorders [6-9]. The medical health workers and especially the frontline workers find themselves exposed to highly risky situations and feel insecure [10] so this may affect the quality of life of the staff. A previous study conducted in “Sotiria” Hospital, in Athens, Greece, found high rates of sleep disturbances among nurse participants and a significant correlation between stress levels and sleep disorders, which affect the quality of life of the hospital staff [11].

Moreover, several studies have lately been conducted worldwide investigating the associations between personality, with the NEO Five Factor Inventory, and the management of the COVID-19 pandemic in personal level [12]. Anglim et al. found that Neuroticism, Extraversion and Conscientious are strong correlates of well-being [13]. The environment (financial resources, information and skills, recreation and leisure, home environment, access to health and social care, physical safety and security, physical environment, transport) may still moderate the relationship between personality and well-being in the general population [14]. Therefore, the aim of this research was to study how the personality traits affect the quality of life of health care workers in a tertiary referral hospital of COVID-19 in Greece, so that interventions can be designed that will contribute to a better quality of staff's life and well-being.

## Materials and methods

Research design

The investigation was carried out at a tertiary referral hospital of COVID-19 in Greece after approval from the Clinical Research Committee (Number 9587/5-4-21). Thoracic Diseases General Hospital "Sotiria" is recognized as the largest pulmonary center in Greece and one of the largest in Europe, with its simultaneous and progressive transformation into a General Hospital after the establishment of Pathology and Surgery clinics. The hospital received the first case of coronavirus in February 2020 and remains until August 2021 a hospital specializing in COVID-19 cases. 

Participants

This study was carried out with total sample of 400 health professionals in public hospital “Sotiria” in Athens, Greece: 69 doctors (17,3%), 211 nurses (52,8%), 57 administrative staff (14,2%), 16 scientific staff (4%) and 47 other (11,8%). Of the participants, 102 were men (25,5%) and 298 were women (74,5%). With regard to age, 76 (19%) were aged between 20 and 30, 122 between 31 and 40 (30,5%), 119 between 41 and 50 (29,8%) and 83 between 51 and 67 (20,8%).

Measures and instruments

The NEO Five Factor Inventory (NEO-FFI) has 60 Likert-type items (12 per domain) ranging from (1=it doesn’t happen to me) to (5=it happens absolutely), derived from the original 240 items [15]. The five factor domains assessed by this measure are neuroticism, extroversion, openness, agreeableness and conscientiousness [15]. The total scores of each subscale are within the range of 0-60. Regarding the reliability in our study, the total Cronbach’s alpha coefficient was = 0.70 for the neuroticism scale = 0.75 for the extroversion scale = 0.77 for the openness scale = 0.64 for the agreeableness scale = 0.74 and for the conscientiousness = 0.72.

The WHOQOL-BREF quality of life assessment was developed by WHOQOL Group [16]. It was administered to measure physical health, psychological health, social relationships and environment. In the WHOQOL-BREF structure is reflected the recognition of the multidimensional nature of quality of life [16]. The WHOQOL-BREF contains a total of 26 questions. Also, one item from each of the 24 facets contained in the WHOQOL-100 has been included and two items from the Overall Quality of Life and General Health facet have been included [16]. The scores of four domains (physical health, psychological health, social relationships and environment) were transformed according to the WHOQOL-BREF manual, to a 0-100 scale [17], with higher domain scores denoting higher QOL. Regarding the reliability in our study, the total Cronbach’s alpha coefficient was = 0.90 for physical health = 0.81 for psychological health = 0.69 for social relationships = 0.78 for environment = 0.80.The NEO-FFI is adapted to the Greek population and the WHOQOL-BREF has been weighted to the Greek population.

Data analysis

Data analysis was conducted using SPSSv.26 and statistical significance was set to 5%. First, the assumption of normality and homocedasticity of variances were checked in order to decide whether to use parametric or non-parametric tests. Results were obtained by means of descriptive statistics, T-test, ANOVA, Pearson’s correlation and regression. The critical level of p<0.05 of Kolmogorov-Smirnov statistics was analyzed, as well as the levels of asymmetry and kurtosis. From these analyses it was concluded that the data did not follow a normal distribution, nevertheless we decided to use parametric tests because the distribution of the data was close to normal distribution.

Procedure

The technique of convenience sampling was followed for the selection of the sample. The sample was recruited from May 2021 to July 2021. The questionnaire was administered in person to the hospital staff. The questionnaire explains both the objectives of the study and the procedures to be followed during the questionnaire, as well as the right to voluntary withdraw from the study if appropriate.

## Results

Of the participants, 102 were men and 298 were women. Most of the sample was between 31 and 40 years old (M = 3.71, SD = 0.52) (30,5%) and the majority were married or partnered (54,5%). 211 people were nurses and the majority worked in the nursing department (Table 1).

**Table 1:** Demographic characteristics of the sample.

Variable	Frequency	Percentage (%)
Gender		
Men	102	25.5%
Women	298	74.5%
Age group		
20-30	76	19%
31-40	122	30.5%
41-50	119	29.8%
>50	83	20.8%
Marital status		
Single	147	36.8%
Married/partnered	218	54.5%
Divorced	34	8.5%
Widow/widower	1	0.3%
Level of education		
Secondary school/lyceum	117	29.3%
University education	175	43.8%
M. Sc. holder/ Ph. D holder	108	27%
Speciality		
Doctor	69	17.3%
Nurse	211	52.8%
Administrative staff	57	14.2%
Scientific staff	16	4%
Other	47	11.8%
Work area		
Nursing department	153	38.3%
Intensive care unit	93	23.3%
Emergency department	44	11%
Accounting department	14	3.5%
Laboratories	34	8.5%
Other	62	15.5%

One-way Anova was utilized to examine the effect of age on quality of life. Age has a significant effect on physical health [F(3,396)=4.12, p=0.007] and the younger ones had the best physical health (Table 2). Moreover, age has a statistically significant effect on psychological health [F(3,396)=3.46, p=0.002] and the younger ones had the highest score in psychological health (M=3.76, SD=0.61) in relation to those over 30 years old. The effect size was average, η2=0.077. Also, age has a significant effect on social relationships [F(3,396)=4.56, p=.004] and the effect size was average, η2=0.063. The younger ones had the highest score in social relationships (M=3.90, SD=0.66) in relation to those over 30 years old. Furthermore, age affects the environment [F(3,396)=3.74, p=0.011] (Table 3).

**Table 2:** Effect of age and marital status on physical health.

	M	SD	One-way Anova	η^2^	p
Age group					
20-30	3.88	0.54	F(3,396)=4.119	0.113	0.007
31-40	3.71	0.52			
41-50	3.62	0.48			
51-67	3.65	0.53			
Marital status					
Single	3.83	0.51	F(3,396)=5.157	0.112	0.002
Married/partnered	3.64	0.51			
Divorced	3.58	0.53			
Widow/widower	3.36	-			

**Table 3:** Effect of age and marital status on the environment.

	M	SD	One-way Anova	η^2^	p
Age group					
20-30	3.42	0.58	F(3,396)=3.744	0.076	0.011
31-40	3.22	0.58			
41-50	3.14	0.60			
51-67	3.16	0.62			
Marital status					
Single	3.33	0.60	F(3,396)=4.240	0.087	0.006
Married/partnered	3.17	0.58			
Divorced	3.15	0.68			
Widow/widower	1.75	-			

Independent sample t-test was utilized to examine the effect of gender on quality of life. Gender has a significant effect on physical health and men had higher score (M=3.79, SD=0.55) in comparison to women (M=3.67, SD=0.50), p=0.04. The effect size was little, d=0.23. Gender has a significant effect on psychological health, with men (M=3.74, SD=0.55) had higher score in comparison with women (M=3.62, SD=0.52), p=0.04. The effect size was little, d=0.22. The specialty, the education level and the work area hadn’t a significant effect on quality of life.

One-way Anova was utilized to examine the effect of marital status on quality of life. Marital status has a statistically significant effect on physical health [F(3,396)=5.157, p=0.002] and the single ones had the highest score in physical health (M=3.83, SD=0.51) in relation to the other marital status (Table 2). The effect size was average, η^2^=0.112. Also, marital status has a significant effect on environment [F(3,396)=4.240, p=0.006] and the effect size was big, η^2^=0.087. Singles had the highest score in environment (M=3.33, SD=0.60) in relation to those with different marital status (Table 3).

The results from the analyzes show that neurotism has a significant negative effect on physical health [β=-0.259, t=-5.765, p=0.001], extroversion has a significant positive effect on physical health [β=0.163, t=2.56, p=0.011], agreeableness has a significant positive effect on physical health [β=0.101, t=2.123, p=0.034] and conscientiousness has a significant positive effect on physical health [β=0.147, t=2.547, p=0.011]. The effect size was average, f2=0.335.

Additionally, the results from the analyzes show that neurotism has a significant negative effect on psychological health [β=-0.290, t=-6.704, p=0.001], extroversion has a significant positive effect on psychological health [β=0.310, t=5.06, p=0.011] and conscientiousness has a significant positive effect on psychological health [β=0.129, t=2.313, p=0.021]. The effect size was average, f2=0.344.

Finally, we found that neurotism has a significant negative effect on social relationships [β=-0.213, t=-4.15, p=0.001], extroversion has a significant positive effect on social relationships [β=0.470, t=6.49, p=0.011] and agreeableness has a significant positive effect on social relationships [β=0.220, t=4.07, p=0.001]. The effect size was big, f2=0.533.

## Discussion

Analyzes have shown that personality traits have a significant effect on quality of life in hospital settings. Especially, we found that neuroticism and extroversion have a significant effect on physical health, psychological health and social relationships, agreeableness affects physical health and social relationships, and conscientiousness has a significant effect on physical health and psychological health. These findings are in agreement with other studies that have found that extraversion is associated with negative effect, during coronavirus crisis and the restrictions measures, because the most extroverted find it difficult to obey restrictive measures. [18,19]. Also, neuroticism is related to the negative estimation of COVID-19 crisis [20-22]. Individuals with higher levels of conscientiousness show stronger COVID-19 caused adaptation [23-25]. Further, a survey study in Canada in early May 2020 found that higher extraversion and lower emotional stability were related to higher perceived stress during the COVID-19 pandemic [26], which may be explained by the fact they are not accustomed to isolation.

With regard to gender differences, our findings are in line with previous studies that reported high levels of stress, anxiety, depression, sleep disturbance and burnout with more frequent and intense symptoms among women [3]. In addition, a research in Greek healthcare professionals has shown that women reported more PTSD symptoms in comparison with men [27]. Also, a study that took place in Italy revealed that being female and not in a relationship was found to be associated with higher depression levels [10]. This finding isn’t in line with our results that shown that marital status had a significant effect only on physical health and environment and not on psychological health.

Moreover, our research highlighted that age affects statistically significant the quality of life and the younger ones are those with better quality. In contrast to this, the results of a research in Spain showed that younger healthcare workers had higher levels of stress, anxiety, depression and post-traumatic stress [28]. One possible explanation for this could be that these workers, have less experience in the field and they aren’t resilient enough in order to handle situations like the COVID-19 pandemic. Research findings show that neuroticism and extroversion are associated with well-being [29] and in the present study neuroticism has a negative effect on physical and psychological health and social relationships perhaps because of the measures of self-protection against COVID-19.

Our study naturally faced some limitations worth mentioning. Firstly, further research is needed to generalize the results with a larger sample from various hospitals treating patients with COVID-19. Secondly, in the present study in the sample women are significantly more than men, as well as scientific staff is very few people (N=160).

## Conclusions

With the present research, we see that personality traits affect a crisis situation with effects on the quality of life of the healthcare workers in a hospital of COVID-19 cases. Based on the research findings we can design and organize therapeutic interventions for the staff, with the aim of improving their quality of life and well-being. Our study hasn’t find a significant effect of specialty, education level and work area on quality of life in a hospital setting. Based on the results, we suggest interventions that deal with psychological pressure, such as the application of mindfulness, enhancing self-care and creation of groups with the hospital staff, where the sharing of emotions and experiences is enhanced, offering one another help, the encouragement and the humor. Finally, the research contributes to a deeper understanding of the impact of personality traits in crisis situations, such as pandemics. 
